# Abracadabra, One Becomes Two: The Importance of Context in Viral −1 Programmed Ribosomal Frameshifting

**DOI:** 10.1128/mbio.02468-21

**Published:** 2022-06-23

**Authors:** Wesley D. Penn, Suchetana Mukhopadhyay

**Affiliations:** a Department of Biology, Indiana University, Bloomington, Indiana, USA; b Department of Chemistry, Indiana University, Bloomington, Indiana, USA; Albert Einstein College of Medicine

**Keywords:** RNA virus, assay development, frameshifting, retroviruses, translational control

## Abstract

The constrained nature of viral genomes has allowed a translational sleight of hand known as −1 Programmed Ribosomal Frameshifting (−1 PRF) to flourish. Numerous studies have sought to tease apart the mechanisms and implications of −1PRF utilizing a few techniques. The dual-luciferase assay and ribosomal profiling have driven the PRF field to make great advances; however, the use of these assays means that the full impact of the genomic and cellular context on −1 PRF is often lost. Here, we discuss how the Minimal Frameshifting Element (MFE) and its constraints can hide contextual effects on −1 PRF. We review how sequence elements proximal to the traditionally defined MFE, such as the coronavirus attenuator sequence, can affect the observed rates of −1 PRF. Further, the MFE-based approach fully obscured −1 PRF in Barley yellow dwarf virus and would render the exploration of −1 PRF difficult in Porcine reproductive and respiratory syndrome virus, Encephalomyocarditis virus, Theiler’s murine encephalomyelitis virus, and Sindbis virus. Finally, we examine how the cellular context of tRNA abundance, miRNAs, and immune response elements can affect −1 PRF. The use of MFE was instrumental in establishing the basic foundations of PRF; however, it has become clear that the contextual impact on −1 PRF is no longer the exception so much as it is the rule and argues for new approaches to study −1PRF that embrace context. We therefore urge our field to expand the strategies and methods used to explore −1 PRF.

## INTRODUCTION

Viruses have evolved multiple ways to maximize the coding capacity of their genome. The idea that one gene gives a single protein fell by the wayside long ago as variant promoters, alternative splicing, and posttranscriptional modifications introduced an entire ecosystem of protein isoforms, but these were behind the curtain, wholesale changes to the protein and mRNA (1–6). Viruses also took a different route, a ribosomal prestidigitation that altered not the mRNA but the ribosomes’ reading frame. Generally, ribosomes are thought of as translating the mRNA 0-frame. However, during translation, it is possible for the ribosome to undertake “translational recoding” by skipping forward or slipping back one to a few nucleotides and entering a new reading frame. This new reading frame will then correspond to the +1, +2, −1, -2 reading frames depending upon the number of skipped/slipped nucleotides. If intentional this recoding is termed Programmed Ribosomal Frameshifting (PRF), and allows viruses to maximize their coding potential by producing variant proteins with altered stoichiometries and potentially, temporal regulation, without changing the coding mRNA (7–10). Yeast and potentially mammalian cells, typically direct ribosomes into −1 frame encoded premature termination codons leading to the rapid degradation of mRNA via Nonsense Mediated Decay (11–13). Thus, in yeast −1 PRF does not expand the genome or alter the stoichiometry so much as it acts to regulate gene expression posttranscriptionally (14). Curiously, the S. cerevisiae Transposable (Ty) elements which, in some ways are analogous to mammalian retroviruses, commonly utilize +1 PRF. Though largely beyond the focus of this review the reader is directed to the outstanding reviews of Farabaugh and Riegger et al. (12, 15).

Initially identified in the Rous Sarcoma Virus, PRF grew from curiosity to crucial with the recognition that −1 PRF controlled Gag-Pol production in HIV-1 (16, 17). These discoveries served as our introduction to a viral sleight of hand which expands the control and repertoire of protein expression possible by highly constrained viral genomes, yeast, prokaryotes, and even mammals (13, 14, 18, 19). While numerous forms of PRF have been described, the most well characterized appears to be −1 PRF which results from interactions between a rapidly translating ribosome with a conserved Minimal Frameshifting Element (MFE). The MFE consists of a heptameric slippery sequence of the form X XXY YYZ, where X are any identical nucleotides, Y is A or U, and Z can be A, C, or U, a spacer region 5 to 9 nucleotides from the slippery sequence followed by either a stem-loop or pseudoknot (20–22). The increased dwell time associated with unwinding the stem-loop/pseudoknot allows the ribosome to slip back a nucleotide thereby changing its reading frame leading to the generation of a unique protein or the premature termination of one (23, 24). While variations on this core architecture have been identified, a substantial body of work, primarily in viruses, has shown that a slippery sequence located the correct distance upstream from a stem-loop or pseudoknot is necessary and sufficient for −1PRFin a multitude of cases (25–28).

Often, the real trick in magic is not the illusion but the distraction that takes you away from a palmed card or hidden rabbit. Thus, for many magic tricks if you want to understand what’s happening, you should look anywhere but where the magician wants you to look or, more directly, context is crucial. The same holds true for −1 PRF, where all too often we focus on the isolated MFE despite numerous examples of how context shapes viral −1 PRF. (Table 1). An overwhelming body of viral −1 PRF research supports the idea that context, be it the mRNA template or the cell itself, is intrinsic to frameshifting utility. Thus, while utilizing the smallest possible fragment of the virus or gene which contains an active frameshifting element (the MFE), has allowed for clear identification of recoding events and even mechanistic dissections, it has also greatly limited our ability to evaluate contextual effects and may limit our ability to understand unique mechanisms, regulation, and outcomes of −1 PRF in viruses, prokaryotes, and eukaryotes.

**TABLE 1:** Non MFE localized effectors of −1 PRF

Type	Virus	Abbreviation	Element	Effect	Distance to Slip Site^*a*^	Could be part of MFE?^*b*^	References
Retro virus	*Human Immunodeficiency Virus 1*	HIV-1	Upstream Sequence/ Ribosomal E-site	Modulates frameshifting	Adjacent	Yes	34, 35
	*Human T-lymphotrophic Virus 2*	HTLV-2	Upstream Sequence	Modulates frameshifting	Adjacent	Yes	34
(+) RNA	*Severe Acute Respiratory Syndrome Coronavirus 2*	SARS-CoV-2	Attenuator Sequence	Decrease apparent frameshifting	~50nt Upstream	Yes	29 – 32
	*Barley Yellow Dwarf Virus*	BYDV	Distal sequence elements	Reinforce the Stem Loop	~3.9 kB Downstream	No	36 – 41
	*Porcine Reproductive and Respiratory Syndrome Virus*	PRRSV	CCCANCUCC motif	Protein binding site, replaces mRNA secondary structure	11nt Downstream	Yes	42 – 48
			nsp1β +PCBP 1/2	Form protein complex, bind mRNA stall translation	~3.5kB Upstream	No	42–48 27, 49–53
	*Encephalomyocarditis Virus*	EMCV	Viral Protein 2A	Reinforce the Stem Loop	~450bp Upstream	No	
	*Theiler's Murine Encephalomyocarditis Virus*	TMEV	Viral Protein 2A	Reinforce the Stem Loop	~410bp Upstream	No/The slippery Sequence is part of the 2a Sequence	27, 49–53 54
	*Sindbis Virus*	SINV	Harrington Motif	Modulate Frameshifting	~200nt Upstream	No	
						

a Distance from the beginning of the sequence element to the beginning of the slippery sequence.

b Based upon general MFE design principles could the MFE be expanded to include this element.

## HOW DO WE EXPERIMENTALLY IDENTIFY AND EVALUATE FRAMESHIFTING?

**FIG 1:**
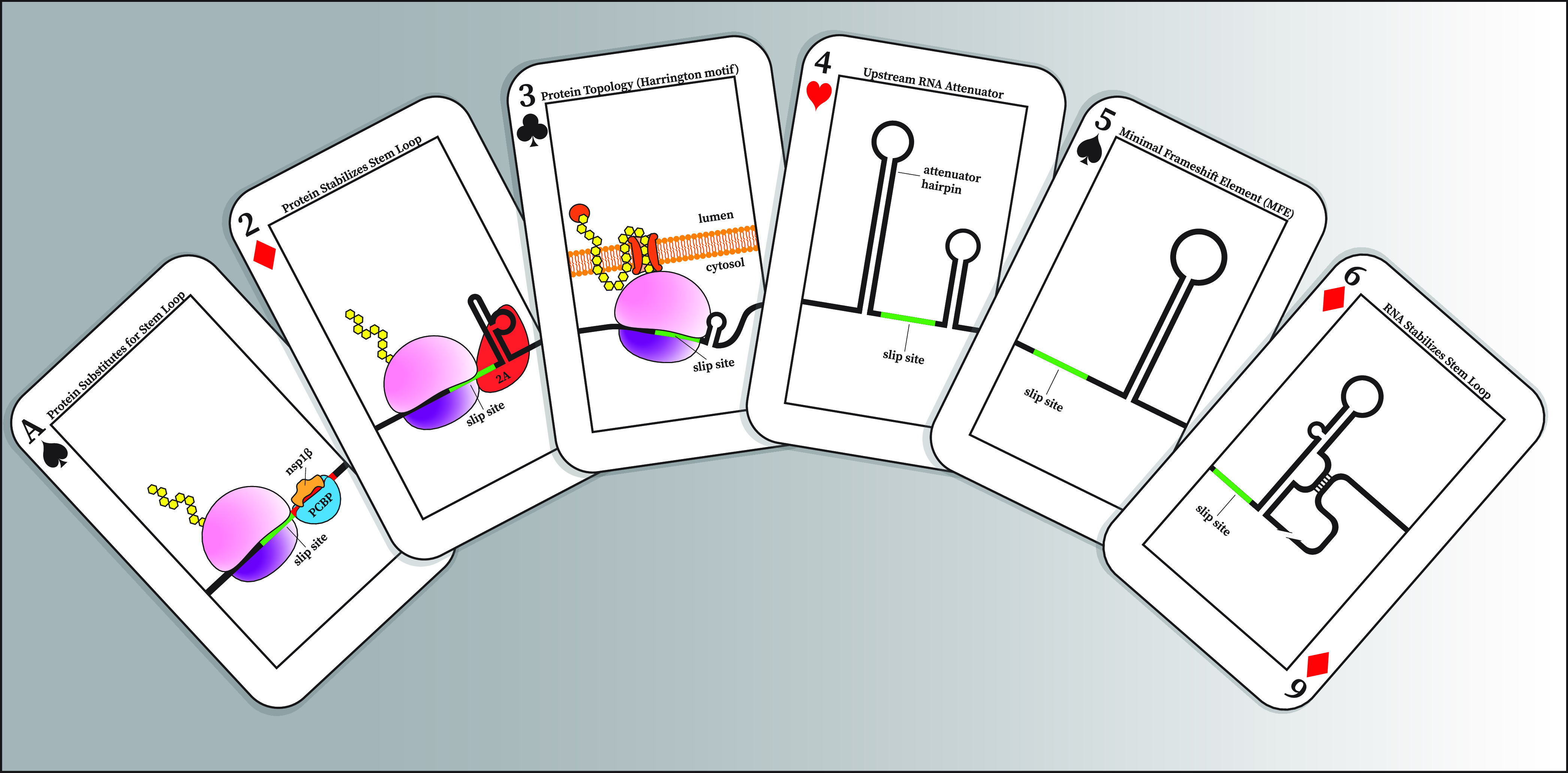
Pick a Mechanism, Any −1 PRF Mechanism. Here, playing cards illustrate some of the contextual elements which affect −1 PRF (not to scale). (A of ♠) The binding of a viral nsp1β/PCBP complex to a sequence element downstream of the slip site in PRRSV. This complex appears to act as a stand in for a stem-loop or pseudoknot. (2 of ♦) For EMCV/TMEV the binding of viral 2A protein reinforces the stem-loop. This leads to an increase in −1 PRF efficiency over time. (3 of ♣) The Harrington Motif. Here, the integration of a marginally hydrophobic TM domain, coupled to the forces of cotranslational folding, modulate −1 PRF efficiency. (4 of ♥) The coronaviridae attenuator sequence seems to disrupt actively translating ribosomes giving the appearance decreased −1 PRF efficiency without actually changing the rate of −1 PRF. (5 of ♠) Comprised of a slippery sequence, spacer region, and stem-loop or pseudoknot the MFE is the base unit of PRF. Individual MFE’s are the most commonly studied unit in −1 PRF. (6 of ♦) Absent stem-loop reinforcement by a sequence element nearly 4Kb downstream of the MFE BYDV is incapable of efficient −1 PRF.

The most common method for measuring PRF utilizes a dual reporter system. These constructs contain a 5′ reporter in frame renilla luciferase followed by the potential PRF element (often the MFE) and then a 3′ out of frame firefly reporter that will only be expressed if PRF occurs (29, 30). Thus, the renilla luciferase provides an approximation of protein production while the firefly luciferase provides an approximation of frameshifting allowing the experimenter to derive a percentage frameshifting value (30). Self-evidently, variations on this theme have proven to be both facile and highly adaptable showing utility in dissecting a range of PRF variants and mechanisms (8). This assay conveniently demonstrates PRF when using the smallest possible region of the target gene or an MFE that is generally in the range of 75 to 100 nucleotides. Larger fragments could take on unique secondary structures or contain elements which mask the effect of PRF. In addition, as the testable element increases in size ribosomes fall off the transcript, collisions occur, or other unanticipated mechanisms alter the rate of synthesis. Additionally, as the MFE is an artificial construct it lacks the full context of viral translation. Within a cell it is the entity of the genome or subgenome which transcriptional machinery interacts with not a small fragment. Thus, the MFE alone is often not enough to induce or accurately characterize frameshifting effects resulting from more distal elements. Therefore, as has been noted, measurements of PRF efficiency in cells can deviate from that seen in isolated experimental systems (31–33) This implies that additional factors, be they viral or host, are playing some role in modulating PRF (Fig. 1).

Ribosomal profiling is rapidly becoming the “gold standard” method used to identify, measure, and understand frameshifting in a manner that is both efficient and independent of artificial MFE constructs. Ribosome profiling also allows for a more discovery-based approach to the identification of −1 PRF locations. Conceptually, ribosome profiling is simply a high throughput version of traditional ribosome footprinting which utilizes the power of next generation sequencing to provide exceptional read coverage (depth) to the sequence of interest (34). Functionally, ribosomes are rapidly stalled during translation and then subjected to an RNase digestion which removes any non-ribosome protected RNA (34). The protecting ribosomes themselves are then removed and the resulting ribosome protected fragments are recovered and subjected to deep sequencing (34). As the work of Michel et al., Napthine et al. and Cook et al. demonstrate, these data can then be mapped onto the target gene/genome allowing one to identify locations where the ribosome may pause for extended periods (increasing numbers of reads), regions where the ribosome is less likely to be found (decreasing number of reads), and even which reading frame the ribosome is likely in (phasing) (35–37). Taken together this information allows the trained eye to easily identify potential PRF positions and, on the whole, is the most accurate method for determining the rate of frameshifting. While this technique is powerful, a limitation is that available ribosome profiling data sets may lack the depth necessary to really show changes in ribosomal occupancy at a given position or the gene of interest lacks this depth. Certainly, in viruses, this is rarely a concern but across the breadth of prokarya and eukarya the problem is more pronounced. Furthermore, ribosome profiling is largely agnostic to mechanism, as while changes in ribosome density across a given transcript or positions can strongly indicate that PRF is occurring at a given location and to what extent, similar to the dual-luciferase assay it does not provide any direct insight into regulation or additional cofactors (37).

The restrictive nature of an MFE means that, absent an experimental discrepancy, −1 PRF can be missed. A striking example of this issue was seen in Barley yellow dwarf virus (BYDV) that produces an RNA-Dependent RNA Polymerase (RDRP) via a -1-frame shift in ORF1 generating ORF2/RDRP (38–40). Having initially identified _BYDV_RDRP as the likely result of a frameshifting event coupled with the identification of classical frameshifting elements in ORF1, the Miller Lab undertook a decade long evaluation of −1 PRF in BYDV (41). Following the traditional approach to studies of −1 PRF, they cloned the BYDV MFE into a plasmid which allowed for the expression of β-glucuronidase (GUS) if a switch to the −1 reading frame occurred (42). Unexpectedly, this construct exhibited a mere 1% frameshifting efficiency (FSE), which in turn was found to be dependent upon the presence of a stop codon (UAG) directly 3′ of the slippery sequence (43). In essence, what they had found then was a classical frameshifting cassette whose mechanism apparently did not match that of other classical cassettes. Further investigating these findings, frameshifting in variant MFE constructs was found to be largely independent of slippery sequence identity but always reliant upon the presence of a stop codon to generate even the relatively modest 1% FSE (43). Conversely, infection with viral RNA provided for an apparent frameshifting rate of 6 to 20% which was independent of the 3′ stop codon but dependent upon slippery sequence identity (43). Taken together, these findings implied a paradox; the classically studied MFE exhibited almost no frameshifting while the virus itself was apparently capable of significant frameshifting to generate _BYDV_RDRP. Intriguingly, the resolution to this paradox forcefully argues for the importance of context in studies of PRF. As Wang et al. had shown that uncapped full-length BYDV mRNA as well as 3′-truncations that included nt4513-5009 were able to produce significant amounts of _BYDV_RDRP, while the work of Paul et al. defined both an element necessary for frameshifting (nt 5046 to 5117) and an enhancer (nt 5118 to 5279) in the BYDV 3′ mRNA (43, 44). Finally, Barry et al. would show that in addition to distal sequence elements, efficient frameshifting required the presence of a stem-loop bulge near the slip site that the distal sequence bound to and presumably reinforced (38). Importantly, none of these elements fit within the traditionally defined MFE structure but do fit with Ziv’s expanded “frameshifting element” model discussed below (45).

Another example of elements that would be missed by using MFE is the coronavirus “attenuator sequence”. a stem-loop ~30 nucleotides in length that ends 2 to 5 nucleotides upstream of the slippery sequence in both SARS-CoV and SARS-CoV-2 (46, 47). The attenuator may cause actively translating ribosomes to stall and drop off the mRNA or act as a gate slowing the rate at which ribosomes traverse the slippery sequence region, thereby giving the appearance of decreased −1 PRF while not directly impacting the actual rate of −1 PRF (46, 48). It is questionable if these attenuator elements would have ever been discovered had Su et al. not wondered why their MFE construct exhibited greater frameshifting than the significantly larger construct utilized by Thiel et al. (~60% versus ~40%) (49, 50). The attenuators’ primary function appears to be stochiometric control, reinforcing the idea that PRF is exquisitely tuned; an individual ribosome that gets past the attenuator will have its potential to frameshift unaltered but fewer ribosomes will encounter the frameshifting element.

Expanding on the attenuator element, Ziv and coworkers have demonstrated that in Coronaviridae and potentially other frameshifting viruses, the functional frameshifting element sits within a much larger ~1.5 kb structure they termed the Frameshifting-Arch (45). Intriguingly they found that the arch is under strong purifying selection and, as such, is one of the most conserved regions of the SARS-CoV-2 genome (45).

One could reasonably argue that as the attenuator sequences do not actually alter the rate of PRF, just the apparent rate, they can safely be ignored in most MFE constructs. However, the work of both Kim et al. and Lèger et al. have shown that the mRNA sequence located directly 5′ to the slippery site can wildly sway the rate of PRF in HIV-1 and HTLV-2 (51, 52). Utilizing a “shuffle” based approach to understand how the sequence surrounding the slippery site and stem-loop effects −1 PRF, Kim et al. were able to demonstrate that the 8 nucleotides preceding the slippery sequence had a modulatory effect on −1 PRF in HIV-1 and HTLV-2 (51). Intriguingly, they found that while the HTLV-2 upstream sequence caused a slight reduction in frameshifting efficiency for HIV-1 (4.5% versus 5.6%) placing the HIV-1 upstream sequence before HTLV-2 led to a nearly 54% increase in frameshifting (14.3% versus 9.3%) (51). Further refining this idea with a focus on HIV-1, Léger et al. provided a possible mechanistic explanation showing that the 3 nucleotide preceding the slippery sequence, those occupied by the ribosomal E-Site upon slipping (A,B, and C in the A-BCX-XXY-YYZ) can increase or decrease the relative rate of frameshifting (52). These findings are largely supported by the recent work of Carmody et al. whose deep mutational scan of the ~300 nucleotide upstream of the slippery sequence in Sindbis virus (SINV) shows that altering the identity of these nucleotides can dramatically impact the observed rate of −1 PRF (53). These findings raise questions then about how MFE design choices could directly impact observed frameshifting efficiencies and mechanisms. While the nucleotides directly upstream of the slippery sequence are unlikely to act as an on/off switch, they are likely to impact the observed rates of frameshifting. Thus, it is entirely possible that dual luciferase, and other MFE-centered assay elements placed too close to the slippery sequence in a given construct could cause significant increases or decreases in observed −1 PRF.

## HOW DO DISTAL ELEMENTS EFFECT −1 PRF?

Although BYDV MFE constructs are largely nonfunctional, they still possess the expected slippery sequence-spacer-stem-loop/pseudoknot architecture that Porcine reproductive and respiratory syndrome virus (PRRSV) appears to lack (35). Indeed, beyond the requisite slippery sequence the only indication that PRRSV may undertake PRF comes from computational analyses showing significant amounts of synonymous site conservation 3′ to the slippery sequence and a striking absence of stop codons in the +1-reading frame: hallmarks of overlapping ORFs (54, 55). In spite of this variance, Fang and coworkers were able to show that not only does PRRSV undertake −1 PRF at this location, rather uniquely, it also utilizes -2 PRF generating an array of nsP2 derived products: nsp2 (no PRF), nsp2N (−1 PRF), and nsp2TF(-2 PRF), that likely play a role in extended wait time coupled to ribosomal movement as well as inhibition of IFN-α expression (55, 56). Mechanistically Li et al. found that PRRSV was able to overcome this lack of a stimulatory mRNA structure through the binding of its nsp1β subunit to a highly conserved CCCANCUCC motif positioned 11 nucleotide downstream of the slippery sequence (57). Indeed, the slippery sequence/CCCANCUCC architecture, though absent in both Equine arteritis virus and Wobbly possum disease virus, is likely the functional unit of PRF in *Arteriviridae* being analogous to the traditional frameshifting cassette (33, 55, 58). Interestingly, while PRRSV seems to “break” the established rules of PRF, it also highlights the incredible regulatory power that such a system can encode. For example, as Cook et al. describe, utilizing the protein product of this *cis* element provides a means of temporal regulation for the virus so that nsp2N/nsp2TF production gradually increases over the course of infection, similar to the behavior seen with EMCV/TMEV (below) (35). In true viral fashion however, PRRSV subverts normal cellular function by hijacking cellular Poly(C) binding proteins 1 and 2 (PCBP 1/2) which then join with nsp1β to form PRF regulatory complexes, wherein PCBP1 primarily affects -2 PRF while PCBP2 primarily affects −1 PRF (27).

Much like BYDV, Encephalomyocarditis virus (EMCV) and Theiler’s murine encephalomyelitis virus (TMEV) exhibit almost no frameshifting when experimentation is limited to the MFE (<1%) (32, 59). However, infection with wild-type virus appears to greatly increase frameshifting efficiency for both EMCV (3.5 to 6.5%) and TMEV (12 to 13%) allowing them to generate more of the frameshifted 2A* (transframe [TF]) protein (32, 59). Computational analysis of the *Cardiovirus* genus identified a region of significant synonymous site conservation between ORFs 2A and 2B corresponding to the StopGo motif that the authors noted was “indicative of functionally important overlapping elements” while the absence of stop codons in the +2/−1-reading frame reinforces this idea (59). Indeed, this region was found to possess both slippery sequences and the secondary mRNA structural elements necessary for −1PRF, albeit with incorrect spacing (59, 60). Experimentation would then show that while the MFE constructs alone were capable of very little −1PRF, coinfection with either WT EMCV or WT TMEV substantially increased the amount of frameshifting observed (32, 59). This implies that some viral-specific element not present in the MFE was key to efficient frameshifting (32, 59). Additionally, the authors were able to determine that frameshifting efficiency increased significantly over time from ~0% at infection to 70% (ribosome profiling) or 46 to 76% (metabolic labeling) at 8 h postinfection in EMCV providing for an unexpected method of temporal regulation (36, 61). Reasoning that the unknown factor might be reinforcing the stem-loop, they were able to utilize the EMCV stem-loop as a bait in the RiboTrap system and identify the viral 2A protein as the unknown factor (62). Interestingly, the authors found that while titration of recombinant 2A protein into either wheat germ extract or rabbit reticulocyte lysate *in vitro* translation systems expressing the EMCV MFE managed a modest 20% frameshifting efficiency, similar TMEV MFE constructs were much more efficient (~58%) in rabbit reticulocyte lysate but similarly modest (~20%) in wheat germ extract (36, 62).

Perhaps the most complex trick yet realized in −1 PRF lies in the recently identified Harrington Motif of the alpha virus genus (63). Defined by a relatively novel architecture this motif is composed of a transmembrane (TM) domain of marginal hydrophobicity situated roughly 45 amino acids upstream of the slippery sequence, thus the TM domain is undergoing cotranslational folding/membrane integration as the ribosome crosses the mRNA slippery sequence (63). Interestingly, the hydrophobicity of this TM domain is such that only occasionally will membrane integration occur. This occasional integration coupled to the forces of cotranslational folding, then generates forces on the ribosome/mRNA sufficient to alter the observed frameshifting rate. Indeed, for SINV, which served to define this motif, −1 PRF appears to occur ~16% of the time driving a switch in protein production from 6K to the TF protein which is reported to be roughly 18% as abundant as 6K (25, 64). Intriguingly, Harrington et al. would show that membrane integration of the relatively polar TM2 of the SINV E2 structural protein occurs ~20% of the time, closely mirroring the observed rates of −1 PRF (63). However, previous work by Chung et al. had shown that devoid of the upstream TM domain a SINV MFE was capable of significant, though less robust, frameshifting (10% versus 16 to 18%) (25). While this finding could imply a lack of correlation between TM integration and −1 PRF, it is more likely an example of how context modifies frameshifting efficiency as Harrington and coworkers further demonstrated that increasing the hydrophobicity of TM2 (i.e., increasing the likelihood of membrane integration) increased the amount of frameshifting some 30% while decreasing the hydrophobicity (i.e., decreasing the likelihood of membrane integration) led to a 61% decrease in frameshifting relative to WT SINV (63). Thus, the work of Harrington et al. shows that while the MFE is necessary and sufficient to demonstrate PRF in most circumstances, it can fail to identify contextual elements that modify the rate of PRF.

## HOW DOES CELLULAR CONTEXT INFLUENCE −1 PRF?

The contextual importance of elements directly encoded by viral mRNA may be somewhat obvious, but the environment or cellular milieu of −1 PRF is tremendously important as well. Indeed, while studying −1 PRF in a cell free or nonnative cellular context may make some measurements cleaner and more robust, it also allows cellular effects to be missed. For example, the most commonly ascribed mechanism of −1 PRF is the “two tRNA” mechanism wherein slipping causes a realignment of both the ribosomal P and A sites from XXY YYZ to XXX YYY despite the associated energetic penalty (for more detailed explanations please see the following references [8, 23, 65–68]). While this appears to be the primary mechanism of −1PRF, work by Rodnina and coworkers have demonstrated that another mechanism termed “one tRNA” or “hungry codon” frameshifting can occur simultaneously with two tRNA mediated −1 PRF (69, 70). Indeed, this finding explains an early observation that ~30% of GagPol proteins generated by HIV-1 have the sequence NFFR which is not accessible through the two tRNA mechanism (17, 70, 71). Here, the localized depletion of a tRNA at the “A”-Site causes a pause in translation long enough for the ribosome to slip backwards thereby producing a unique sequence (70). This demonstrates how the specific cellular context of a frameshifting signal, such as the relative abundances of various tRNA species which can vary between cell types, can determine the mechanism by which −1 PRF occurs and its effects.

Though not directly viral in nature itself, a most intriguing example of −1 PRF regulated gene expression comes from human CCR5. Predominantly expressed by immune cells, CCR5 is a co-receptor for HIV-1 entry into CD4^+^ T-Cells that computational analysis by Dinman and colleagues identified as potentially subject to −1 PRF (11, 13). Surprisingly, given −1 PRF’s role in retroviral replication and some 5 to 8% of the human genome containing retroviral derived gag and pol genes, only a few functional −1 PRF sites have been identified in mammalian genomes (18, 72–76). Thus, Belew and coworkers experimentally demonstrating cell dependent frameshifting of CCR5 in the range of 4.5%−11%; roughly equivalent to the ~15% frameshifting seen in ribosome profiling data, was remarkable (13). Furthermore, this work reinforced the role of context in −1 PRF as the authors were able to show that microRNA miR-1224 stimulated −1 PRF of CCR5 in a dose dependent manner independent of HIV-1 infection (13). In turn, this increased frameshifting was found to decrease both CCR5 mRNA and protein abundance through non-sense mediated decay mechanisms, implying that these effects could be part of an antiviral response by an infected cell (13). (Note: While this paper was under revision Khan et al. published a paper calling into question these findings [77]).

As previously discussed, a key function of −1 PRF appears to be the maintenance of relatively tight stochiometric ratios during viral replication as both increases and decreases in the rate of frameshifting can be deleterious to viral fitness. Indeed, this dependency has provided a unique target for the development of several small molecule antiviral compounds that have shown promising results *in vitro* (78). Yet, as is so often the case, evolution appears to have beaten us to the punch in the form of Shiftless (SHFL, SFL, C19orf66, RyDEN, IRAV) an interferon stimulated gene that itself acts to inhibit −1 PRF and, thereby, viral replication (79–81). Identified via a screen of interferon stimulated genes, SHFL was initially found to inhibit the infection/replication of all known human dengue virus serotypes through an unknown translation suppressive mechanism (79). Importantly, additional work has shown that a range of RNA viruses, including SARS-CoV-2, DNA viruses, and other −1 PRF elements are susceptible to SHFL mediated inhibition (79, 81–84). Interestingly, SHFL also appears to aid in the degradation of Zika and Japanese Encephalitis virus NS3 through lysosomal degradation (82, 84). Subsequent work by Wang et al. connected these observations to the observed −1 PRF suppressive effects of SHFL in a landmark paper evaluating the regulation of HIV-1 Gag-Pol expression (81). Unexpectedly, they found that SHFL appears to preferentially bind mRNA containing an intact −1 PRF signal (81). However, a recent report by Napthine et al. argues for a more generalized mode of RNA binding by SHFL in a manner reminiscent of UPF1 which it has been shown to interact with (80, 83, 85). Regardless of specific binding propensity, SHFL expression leads to an ~33% decrease in the observed rate of frameshifting for HIV-1 and ~77% for TMEV (81, 83). Functionally, SHFL appears to drive the dissociation of ribosomes traversing the PRF cassette through increased pausing of noncanonical rotated states, thereby inducing a stall on the slippery sequence (81, 83). This in turn leads to the production of premature termination products via the action of eRF1:eRF3 to release the stalled ribosome thus providing a concomitant decrease in frameshifting (81, 83). Or, in simpler terms, SHFL causes the ribosome to become stuck and then removed from the slippery sequence preventing production of the frameshifted product.

Beyond regulating the relative rate of −1 PRF, cellular context can also dictate the mechanism of frameshifting. As elucidated by Korniy et al., tRNA abundance can drive −1 PRF from the more common two-tRNA mechanism to the less common one-tRNA or “hungry codon” mechanism (70, 86). Indeed, this environmental factor explains the curious observation that −1 PRF in HIV-1 produces two products which differ by a single amino acid insertion at a ratio of approximately 70:30 (17, 71). Broadly speaking, in the two-tRNA mechanism frameshifting occurs at the slippery sequence when correctly paired (codon:anticodon) tRNA’s occupying the ribosomal A and P sites transition into a new, less energetically favorable, codon:anticodon pairing. Though the exact mechanism of this transition is still in question for many viruses, a combination of increased dwell time at the slippery sequence combined with unwinding of the downstream mRNA secondary structure appears to play some role (23, 65, 67, 87). Indeed cryo-EM structures of SARS-CoV-2 produced by Bhatt et al. largely seem to reinforce these previous findings (88). Here, the authors find that the ribosome likely pauses for an extended period of time at the post slippery sequence pseudoknot so that the ribosomal P-site aligns with the −1 frame. This differs from the A-site mRNA rearrangement seen in HIV-1. Conversely, in one-tRNA frameshifting local depletion of a given tRNA at the ribosome A site leads to extended pausing as the ribosome awaits delivery of the correct tRNA (70, 86). Hence, the combination of extended wait time coupled to ribosomal movement, cause the ribosome to slip back a single nucleotide allowing for the incorporation of a more abundant tRNA and in turn recoding (70). Thus, the specific cellular context of tRNA abundance can affect the mechanism of PRF. In summation then, we must consider cellular context when choosing how to conduct our experiments and we must be aware of how these choices, as in all cases, can bias our results. Studying a PRF signal in a nonnative context can influence what miRNA’s, tRNAs, and assorted cellular cofactors may be present are present thereby impacting the observed rates of −1PRF.

## DIRECTIONS TO GO MOVING FORWARD

The point of this review has been to demonstrate that a growing body of research shows that context is exceedingly important to the understanding of −1 PRF, and to move forward as a field we must expand our repertoire to include these elements to determine the mechanism of PRF. The reductionist approach of the dual-luciferase assay’s MFE and the mechanistically agnostic ribosomal profiling have repeatedly proven their utility in identifying and evaluating potential PRF sites, we must also concede that these approaches are underpowered to fully explore how context tunes PRF. To our mind, using the dual-luciferase assay and ribosomal profiling to identify and roughly characterize −1 PRF sites should be seen as a starting point. Further characterization should focus on how the interplay between the frameshifting cassette, mRNA elements, proteins, and the host cell can increase or decrease PRF. No longer should we view −1 PRF in binary terms. We now know that PRF is functionally a continuum whose relative rates are often greatly influenced by elements outside the MFE.

We suggest as we move forward it is incumbent upon those in this multidisciplinary field to redefine the MFE so that it includes those positions directly upstream of the slippery sequence which have been shown to play a significant role in modulating −1 PRF such as the E site codon and attenuator sequence. Furthermore, finding a way to reliably screen for −1 PRF effectors outside the MFE which accounts for the effects of ribosomal collisions and pileups is central to a more holistic approach to studies of −1 PRF. Therefore, successfully unraveling the mechanisms and actions of −1 PRF across the kingdoms of life will require a range of techniques, including, variations on the dual-luciferase assay as well as ribosome profiling. Indeed, no magician or illusionist relies on a few tricks, they instead build their performances through the creative combination of a few different basic techniques and skills which combine to create “magical” results, an idea which could also be applied to the study of −1 PRF.

Admittedly, expanding studies of −1 PRF to routinely consider context is likely to be challenging as the design of experiments which probe −1 PFR is often limited by technical considerations and the tradeoffs inherent in many controls. While we understand that expanding the experimental toolkit and taking a more context inclusive the approach to studies of −1 PRF are unlikely to occur overnight we hope that moving forward our field will begin to adopt strategies which look beyond the MFE to the greater secrets of −1 PRF.
